# Structure-based virtual screening for PDL1 dimerizers: Evaluating generic scoring functions

**DOI:** 10.1016/j.crstbi.2022.06.002

**Published:** 2022-06-09

**Authors:** Viet-Khoa Tran-Nguyen, Saw Simeon, Muhammad Junaid, Pedro J. Ballester

**Affiliations:** a Centre de Recherche en Cancérologie de Marseille (CRCM), Inserm, U1068, Marseille, F-13009, France; b CNRS, UMR7258, Marseille, F-13009, France; c Institut Paoli-Calmettes, Marseille, F-13009, France; d Aix-Marseille University, UM 105, F-13284, Marseille, France

## Abstract

The interaction between PD1 and its ligand PDL1 has been shown to render tumor cells resistant to apoptosis and promote tumor progression. An innovative mechanism to inhibit the PD1/PDL1 interaction is PDL1 dimerization induced by small-molecule PDL1 binders. Structure-based virtual screening is a promising approach to discovering such small-molecule PD1/PDL1 inhibitors. Here we investigate which type of generic scoring functions is most suitable to tackle this problem. We consider CNN-Score, an ensemble of convolutional neural networks, as the representative of machine-learning scoring functions. We also evaluate Smina, a commonly used classical scoring function, and IFP, a top structural fingerprint similarity scoring function. These three types of scoring functions were evaluated on two test sets sharing the same set of small-molecule PD1/PDL1 inhibitors, but using different types of inactives: either true inactives (molecules with no *in vitro* PD1/PDL1 inhibition activity) or assumed inactives (property-matched decoy molecules generated from each active). On both test sets, CNN-Score performed much better than Smina, which in turn strongly outperformed IFP. The fact that the latter was the case, despite precluding any possibility of exploiting decoy bias, demonstrates the predictive value of CNN-Score for PDL1. These results suggest that re-scoring Smina-docked molecules with CNN-Score is a promising structure-based virtual screening method to discover new small-molecule inhibitors of this therapeutic target.

The protein-protein interaction of Programmed Cell Death-1/Programmed Cell Death-Ligand 1 (PD1/PDL1) leads to tumor resistance to apoptosis and promotes tumor progression. It has become one of the most promising targets for cancer immunotherapy, as inhibiting this interaction helps prevent its pro-tumor activity. The U.S. Food and Drug Administration has approved seven PD1/PDL1-targeting monoclonal antibody therapies, four binding to PD1 and the others to PDL1 receptors, providing effective PD1/PDL1 blockades (Upadhaya et al., 2022). There is currently a strong interest in finding small-molecule PD1/PDL1 modulators to overcome the disadvantages of therapeutic antibodies, e.g. immune-related side effects, the lack of oral bioavailability and high treatment prices (Konieczny et al., 2020). An innovative mechanism to disrupt PD1/PDL1 is via PDL1 dimerization (Shi et al., 2019), relying on a small molecule that binds to PDL1, inducing the formation of a PDL1 dimer and therefore impeding its interaction with PD1. There is thus a need for *in silico* models that can facilitate the search for potent PDL1 dimerizers.

We hypothesize that generic machine-learning (ML) scoring functions (SFs) would be an effective manner to carry out structure-based (SB) virtual screening (VS) for PDL1 dimerizers. This is based on numerous studies reporting the superiority of these multi-target-trained MLSFs (Shen et al., 2019; Li et al., 2020; Adeshina et al., 2020; Fresnais and Ballester, 2020; Nguyen et al., 2019; Li et al., 2021; Durrant et al., 2015; Meng and Xia, 2021; S á nchez-Cruz et al., 2021), including those that reported the discovery of potent actives for various other targets (Adeshina et al., 2020; Durrant et al., 2015; Ghislat et al., 2021). With this purpose, we evaluated an MLSF based on convolutional neural network (CNN) models called CNN-Score (Ragoza et al., 2017), which is an ensemble of five CNN models (deep learning architecture with 7–20 hidden network layers) selected to balance pose prediction, VS performance and runtime. Only two models (dense and default2017) were trained using a large proportion of property-matched decoys (assumed inactives) extracted from the DUD-E database (1,429,790 training instances per model, including 22,645 docked active instances). By contrast, the other three (general_default2018, crossdock_default2018, redock_default2018) were entirely trained on experimental data from PDBbind (11,324, 13,839, and 13,780 instances, respectively). It is noteworthy that PD1/PDL1 is not one of the 102 targets of DUD-E and, among all PDBbind-extracted training instances, only two correspond to a dimer or a tetramer (PDB IDs: 5N2F, 5N2D) of PDL1 co-crystallized with a small-molecule PD1/PDL1 inhibitor. In this study, the performance of two non-MLSFs was also evaluated on the same data and compared to that of CNN-Score. The first of these is Smina (Koes et al., 2013), a classical SF employed by the docking program of the same name that was used to dock all test molecules. The second is IFP (Tran-Nguyen et al., 2021), measuring the similarity of ligand-target complexes by their interaction fingerprints. IFP was recently reported as a superior SF in retrospective SBVS experiments on other targets, outperforming GRIM and two generic MLSFs (Tran-Nguyen et al., 2021).

The experimental design of this study is illustrated in Fig. 1. Small-molecule PDL1 dimerizers (true actives) were first mined from known patent data, while true inactives were retrieved from PubChem BioAssay (step 1). Assumed inactives (property-matched decoys) were generated by DeepCoy (Imrie et al., 2021) for each active (step 2), before all molecules were docked into the PDL1 template with Smina (step 3). The resulting ligand-receptor complexes were scored using the three aforementioned SFs (step 4), after which their performance was evaluated and compared (step 5).

**Fig. 1:**
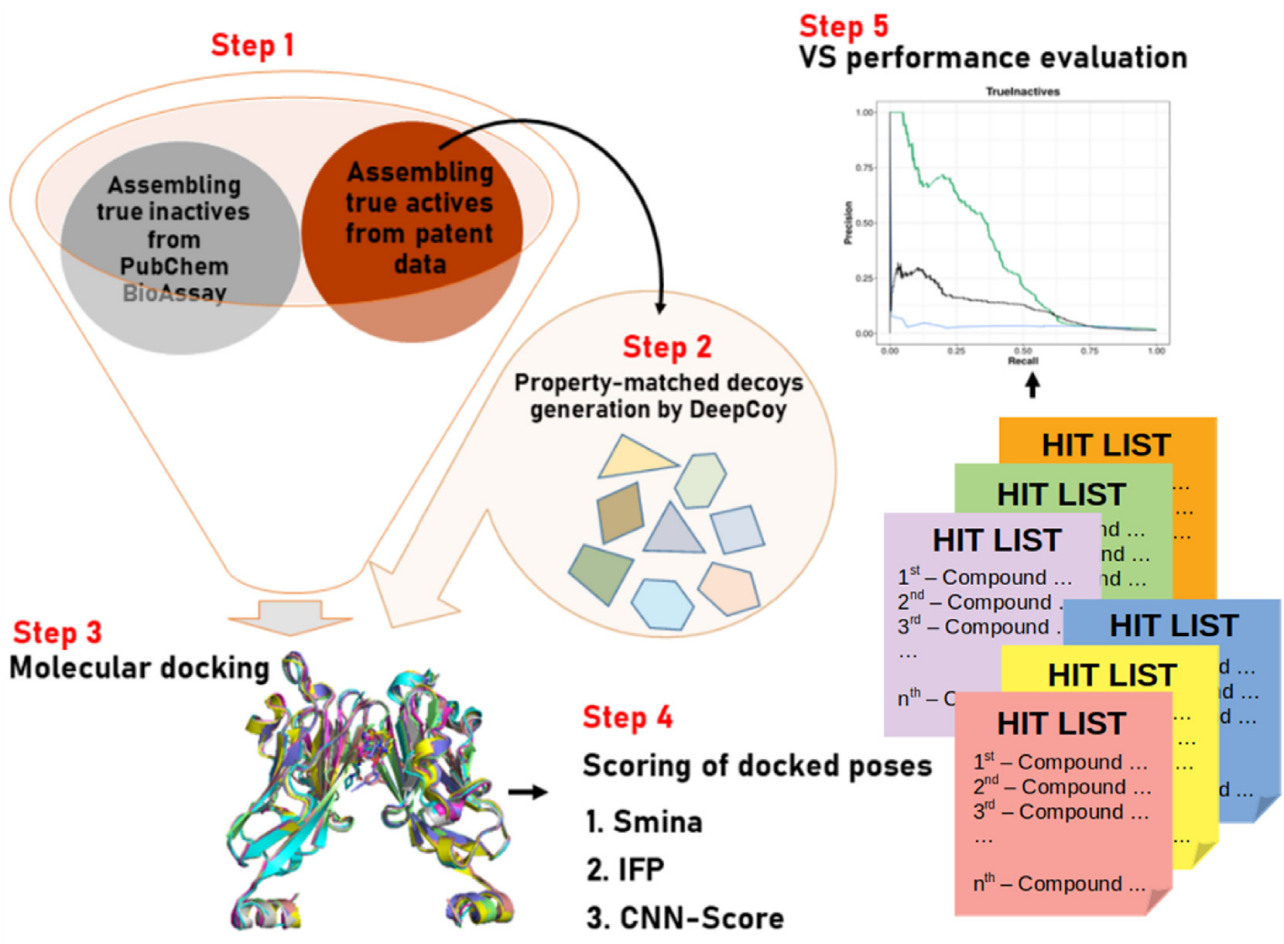
Overview of the experimental design to evaluate three generic SFs for SBVS against PDL1.

The use of PDL1 dimers to dock PD1/PDL1 inhibitors has been observed in the literature, and was proven promising for SBVS in a recently published pilot study (Kuang et al., 2020). In the same vein, we chose the 6NM8 PDB structure as the PDL1 template for this study due to the following reasons: (i) it is a PDL1 homodimer co-crystallized with a known small-molecule PD1/PDL1 inhibitor (HET code: KSD), (ii) the structure was resolved by X-ray diffraction crystallography and is of acceptable resolution (2.79 ​Å), and (iii) the ligand KSD binds with PDL1 in a non-covalent manner on the same interface as that observed in PD1/PDL1 complexes (Fig. 2). The structure of the target was downloaded from the Protein Data Bank and prepared using the DockPrep tool of Chimera (v.1.15) with its recommended parameters. Standard preparation workflows for protein structures were performed: solvent molecules and non-complexed ions were deleted, while hydrogen atoms and charges were added at pH 7.4.

**Fig. 2:**
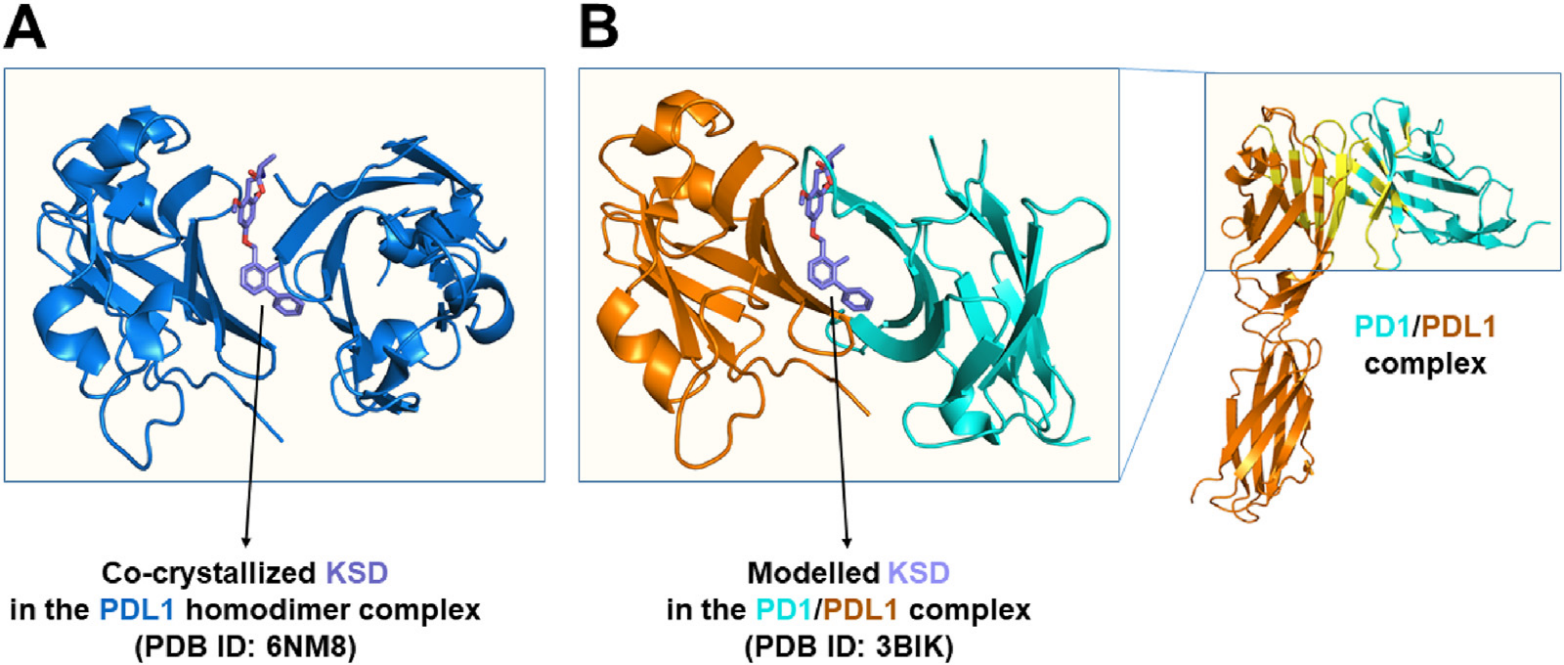
(**A**) Homodimeric structure of PDL1 (PDB ID: 6NM8) showing that the co-crystallized ligand (HET code: KSD) binds to PDL1 (**B**) on the same interface as that observed in PD1/PDL1 complexes (zoomed view from the top of the PD1/PDL1 region in the full structure).

Two test sets were constructed to compare how well each SF would rank the same set of docked actives embedded in a much larger pool of docked inactives. The same 296 PDL1 dimerizers, retrieved from the patent WO2015034820A1, act as true actives in both test sets (KSD does not form part of the test sets). The first test set is called TrueInactives, as its inactive components come from the *in vitro* PubChem assay ID 2316, which only identified a single weak active, denoting PD1/PDL1 as a hard target. The second test set is named DeepCoys, containing DeepCoy-generated structurally-dissimilar molecules having similar physico-chemical properties to the actives. A novel method based on graph-generative neural networks, DeepCoy was shown to offer more challenging decoys in VS scenarios than property-matched decoys employed in DUD-E and DEKOIS, while posing no additional risk of false negative bias in the resulting data set, according to its original study (Imrie et al., 2021). 14,650 decoys were generated for this test set, corresponding to 50 decoys per active (three actives out of 296 were not processed by DeepCoy).

Each molecule was first prepared from its SMILES string using the ChemAxon suite (v.21.18.0) as follows: removal of inorganics and mixtures, 3D structural conversion and cleaning, normalization of specific chemotypes, and removal of duplicates. A 3D sdf structure was issued for each ligand. The Open Babel software (v.2.3.1) was next used to prepare 3D mol2 files from the above sdf: protonation was done on an atom-by-atom basis, while MMFF94 partial charges were assigned, such that molecules with multiple ionizable centers would have all centers ionized. The final output files were ready for the subsequent docking procedure.

The Smina software (v.2019-10-15) was used to carry out molecular docking. The search space was centered on the co-crystallized ligand of 6NM8, and the size of each axis was set at 30 ​Å to give the ligands sufficient space to rotate. Only one pose was output per docked molecule. Note that there is so far no strict rule as to how many docked poses should be retained per ligand for re-scoring (single-pose scoring or multi-pose scoring); and the difference, if there were any, in VS performance that results from these two approaches depends largely on the target and the screened data. All output poses were saved as mol2 files. The native Smina generic SF also predicted the binding free energy for each ligand.

The IFP module from the IChem package (v.5.2.9) was used to compute the Tanimoto score between the IFP of each docked pose and that of the crystal ligand KSD (the higher the score, the better the molecule). The 3D structures (mol2) of the receptor, the crystal ligand, and each docked pose are required to calculate the corresponding IFPs, on the basis of all receptor residues engaging in direct interactions with the small molecules, taking into account the following features per ligand: hydrophobic, aromatic, hydrogen-bond, and electrostatic.

The Smina scores, the IFP Tanimoto similarity scores, and the CNN-Scores were used to rank all docked molecules, generating, in total, six hit lists corresponding to our two test sets and three generic SFs. Precision-recall (PR) areas under the curve (AUCs) were computed using the scikit-learn toolkit (v.1.0.2). PR curves are useful for assessing VS performance when the classes are highly imbalanced, as in this case.

Fig. 3 portrays the performance of Smina, IFP, and CNN-Score on the same TrueInactives and DeepCoys test sets by means of PR curves. On the TrueInactives set, CNN-Score (PR-AUC ​= ​0.33) performed much better than Smina (PR-AUC ​= ​0.12), which, in turn, strongly outperformed IFP (PR-AUC ​= ​0.04). This trend was also observed when the same PDL1 actives were distinguished from the property-matched decoys in the DeepCoys test set (PR-AUCs of CNN-Score, Smina, and IFP equal to 0.53, 0.08, and 0.04, respectively). Despite the poor discriminatory power of the two non-MLSFs evaluated in this study (Smina, IFP), their performance on both test sets was still at least twice better than it would be expected from random guessing (PR-AUC ​= ​0.01 for the TrueInactives set and 0.02 for the DeepCoys set, which are given by P/(P ​+ ​N) (Saito and Rehmsmeier, 2015), with P being the number of positives or actives, and N being the number of negatives or inactives).

**Fig. 3:**
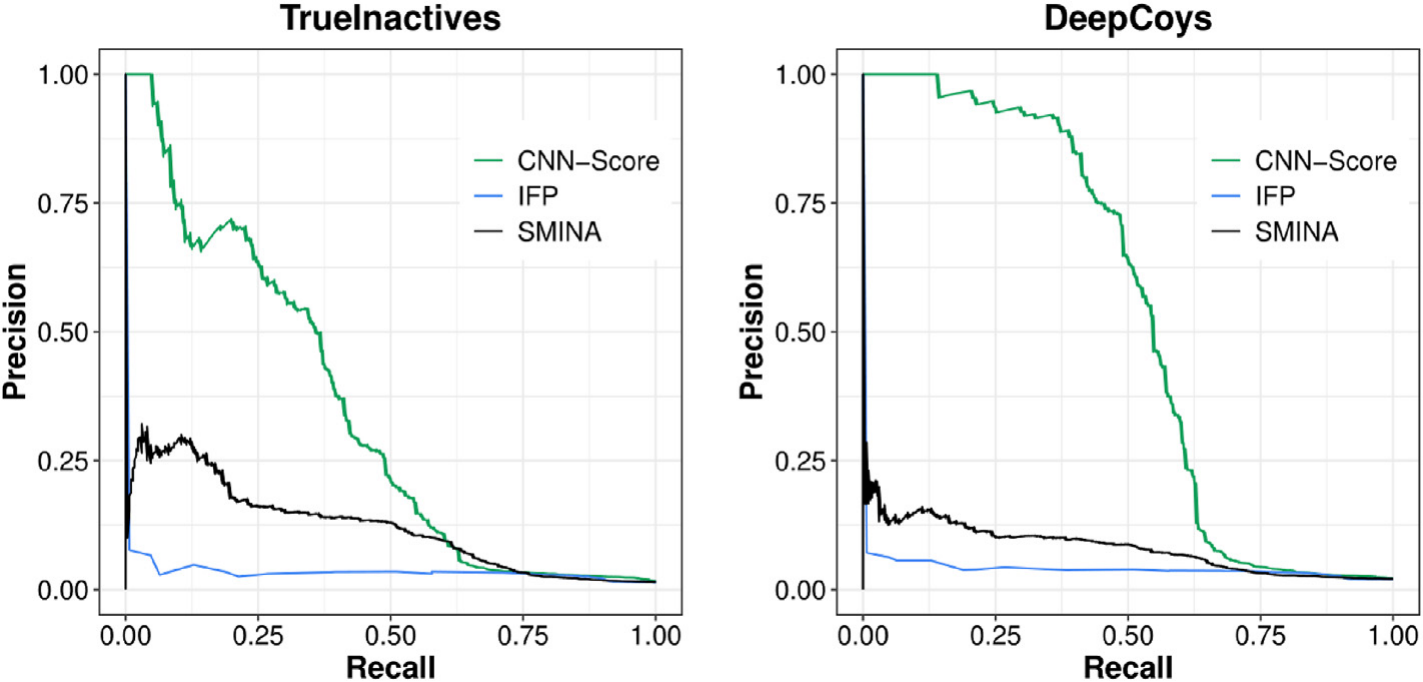
SBVS performance of generic SFs portrayed by PR curves on two test sets. The three generic SFs Smina, IFP, CNN-Score were tested on the same TrueInactives and DeepCoys test sets. Their PR curves are portrayed in black (Smina), in blue (IFP), and in green (CNN-Score). (For interpretation of the references to colour in this figure legend, the reader is referred to the Web version of this article.)

IFP was observed to perform poorly on both test sets. On some other targets, IFP was reported to outperform two MLSFs namely Pafnucy and Δ_vina_RF_20_ (Tran-Nguyen et al., 2021). However, these MLSFs were trained on data not including any true or assumed inactives (Tran-Nguyen et al., 2021), which is known to be suboptimal for SBVS (Wójcikowski et al., 2017) and hence partly the reason why their SBVS performance was inferior to that of IFP. Here we found that IFP performed on PDL1 much worse than CNN-Score or even Smina. This interaction-based SF ranks docked molecules according to how similar they are to a reference, in terms of interaction mode with the receptor. It may easily identify actives with similar scaffolds and receptor-binding modes to those of the reference, but may fail to do so if otherwise, due to the dissimilarity of interaction fingerprints. Fig. 4 illustrates this point: the co-crystallized ligand of PDL1 and a true PDL1 dimerizer (1–71) are shown inside their receptor. The orientation of the two molecules is different, hence their interaction modes with the protein. As a result, the Tanimoto similarity of their receptor-interacting fingerprints is relatively low: IFP did not retrieve this true active among the top 1%-ranked molecules on both test sets, while CNN-Score did. Other authors have also observed that MLSFs outperformed IFP on many other targets (Shen et al., 2021) (the difference is larger with target-specific MLSFs, which were not considered in the original study (Tran-Nguyen et al., 2021)). Another point concerns the choice of a reference ligand to run IFP (Tran-Nguyen et al., 2021): the proportion of screened actives similar to the reference may affect ligand-based and interaction-based VS (Ballester et al., 2009), and this is only known in retrospective settings.

**Fig. 4:**
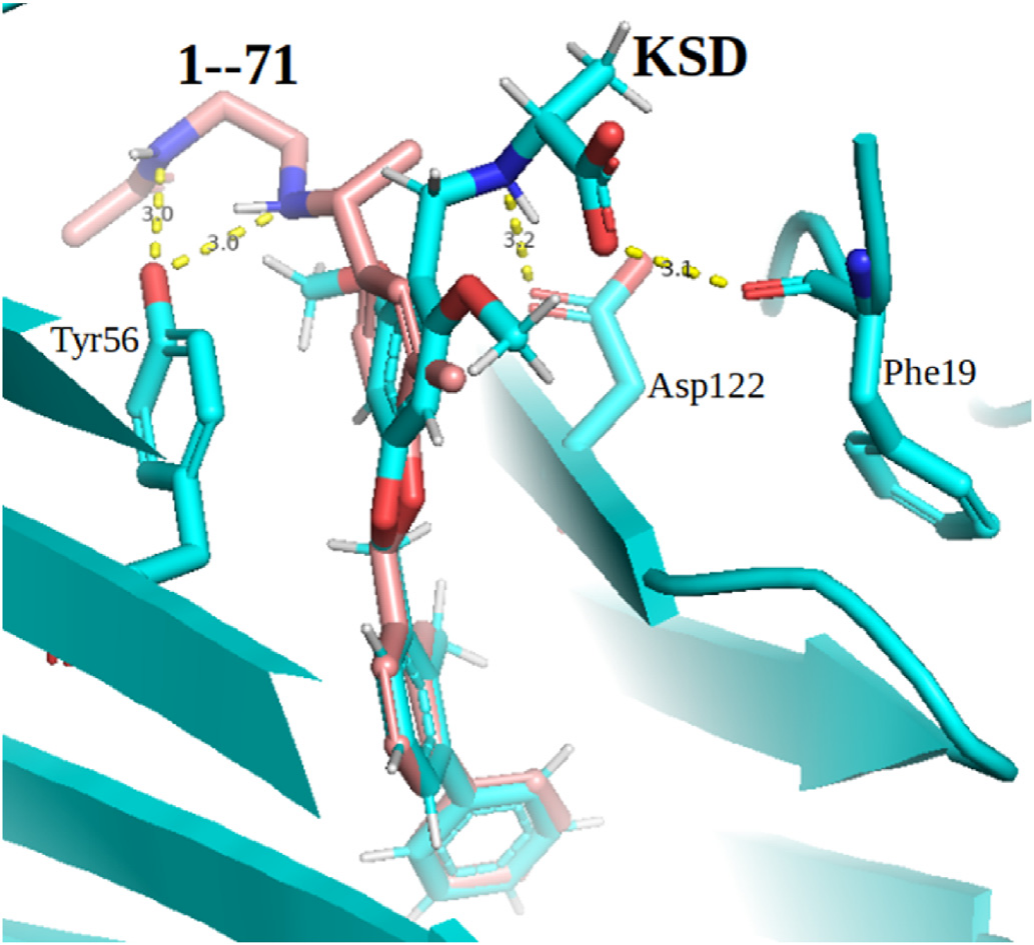
The co-crystallized ligand of PDL1 (KSD in its crystallographic pose, labeled, in cyan) and a true PDL1 dimerizer (1–71 in its top-ranked pose generated by Smina, labeled, in light red) inside their receptor. While KSD forms two hydrogen bonds with Phe19 and Asp122, the active 1–71 forms two hydrogen bonds with Tyr56. Their receptor-interacting fingerprints are therefore dissimilar, and IFP failed to retrieve this true active among the top 1%-ranked molecules on both test sets, while CNN-Score managed to do so. (For interpretation of the references to colour in this figure legend, the reader is referred to the Web version of this article.)

CNN-Score performance is influenced by the test decoys. As explained before, this SF was partly trained on DUD-E decoys whose physico-chemical properties were matched to those of training actives (W ó jcikowski et al., 2017). DeepCoys decoys are also property-matched to an active in a similar way. Since the performance of an MLSF is overestimated when it is trained and tested with the same type of property-matched decoys, it is likely that CNN-Score was somewhat overestimated on the DeepCoys test set. However, there is no reason to think that its performance only comes from exploiting decoy bias: multiple examples suggest that this is actually far from being the case (Ballester, 2020). Here we found yet another example: CNN-Score performance on TrueInactives, where no decoy bias is exploited, was still much better than that of Smina and IFP. As the nature of the inactives (true or decoy) is the only factor that varies between our two test sets, decoy bias must be responsible for the difference in the PR-AUCs of CNN-Score (0.53 versus 0.33). Nevertheless, the performance of this SF is unlikely affected by docked pose quality: its discriminatory power did not vary when only one best-ranked pose or multiple poses were retained per ligand for scoring, according to its original paper (Ragoza et al., 2017). Besides, the chemical structures of our test actives are diverse (Supporting Information): there was hence no favoritism towards certain scaffolds that might lead to the SF's good performance.

To conclude, the results of this study suggest that CNN-Score is a promising SBVS method to discover PDL1 dimerizers after chemolibraries are docked to the 6NM8 structure of PDL1. It is remarkable that only three out of five models constituting the CNN-Score architecture were trained on data involving PDL1, and that such data only represent a tiny proportion of those used to train each of these models (below 0.02%), therefore implying that its predictive accuracy on PD1/PDL1 mainly comes from exploiting data related to other targets. In the future, we plan to investigate MLSFs trained on both experimental and synthetically-generated PDL1 data, as such target-specific approaches generally result in improved predictive accuracy (Fresnais and Ballester, 2020; W ó jcikowski et al., 2017; Xiong et al., 2020; Yasuo and Sekijima, 2019).

## Code availability

Code and processed datasets to reproduce the results in this paper are freely available at https://github.com/sawsimeon/PDL1_Generic.

## CRediT authorship contribution statement

**Viet-Khoa Tran-Nguyen:** Writing – original draft. **Pedro J. Ballester:** Supervision.

## Declaration of competing interest

The authors declare the following financial interests/personal relationships which may be considered as potential competing interests:

Viet-Khoa Tran-Nguyen reports financial support was provided by 10.13039/501100004097Fondation ARC pour la Recherche sur le Cancer (ARC). Pedro J. Ballester reports financial support was provided by 10.13039/501100001852Indo-French Centre for the Promotion of Advanced Research. Pedro J. Ballester reports financial support was provided by 10.13039/501100001665French National Research Agency. Pedro J. Ballester reports financial support was provided by 10.13039/501100004097Fondation ARC pour la Recherche sur le Cancer (ARC).

## References

[bib1] Adeshina Y.O., Deeds E.J., Karanicolas J. (2020). Machine learning classification can reduce false positives in structure-based virtual screening. Proc. Natl. Acad. Sci. Unit. States Am..

[bib2] Ballester P.J. (2020). Selecting machine-learning scoring functions for structure-based virtual screening. Drug Discov. Today Technol..

[bib3] Ballester P.J., Finn P.W., Richards W.G. (2009). Ultrafast shape recognition : evaluating a new ligand-based virtual screening technology. J. Mol. Graph. Model..

[bib4] Durrant J.D. (2015). Neural-network scoring functions identify structurally novel estrogen-receptor ligands. J. Chem. Inf. Model..

[bib5] Fresnais L., Ballester P.J. (2020). The impact of compound library size on the performance of scoring functions for structure-based virtual screening. *Brief. Bioinform.* bbaa095.

[bib6] Ghislat G., Rahman T., Ballester P.J. (2021). Recent progress on the prospective application of machine learning to structure-based virtual screening. Curr. Opin. Chem. Biol..

[bib7] Imrie F., Bradley A.R., Deane C.M. (2021). Generating property-matched decoy molecules using deep learning. Bioinf..

[bib8] Koes D.R., Baumgartner M.P., Camacho C.J. (2013). Lessons learned in empirical scoring with smina from the CSAR 2011 benchmarking exercise. J. Chem. Inf. Model..

[bib9] Konieczny M. (2020). Di-bromo-Based small-molecule inhibitors of the PD-1/PD-L1 immune checkpoint. J. Med. Chem..

[bib10] Kuang Z. (2020). Partial least-squares discriminant analysis and ensemble-based flexible docking of PD-1/PD-L1 inhibitors: a pilot study. ACS Omega.

[bib11] Li H., Sze K.-H., Lu G., Ballester P.J. (2020). Machine-learning scoring functions for structure-based drug lead optimization. Wiley Interdiscip. Rev. Comput. Mol. Sci..

[bib12] Li H., Sze K.-H., Lu G., Ballester P.J. (2021). Machine-learning scoring functions for structure-based virtual screening. WIREs Comput. Mol. Sci..

[bib13] Meng Z., Xia K. (2021). Persistent spectral-based machine learning (PerSpect ML) for protein-ligand binding affinity prediction. Sci. Adv..

[bib14] Nguyen D.D. (2019). Mathematical deep learning for pose and binding affinity prediction and ranking in D3R Grand Challenges. J. Comput. Aided Mol. Des..

[bib15] Ragoza M., Hochuli J., Idrobo E., Sunseri J., Koes D.R. (2017). Protein–ligand scoring with convolutional neural networks. J. Chem. Inf. Model..

[bib16] Sánchez-Cruz N., Medina-Franco J.L., Mestres J., Barril X. (2021). Extended connectivity interaction features: improving binding affinity prediction through chemical description. Bioinformatics.

[bib17] Saito T., Rehmsmeier M. (2015). The precision-recall plot is more informative than the ROC plot when evaluating binary classifiers on imbalanced datasets. PLoS One.

[bib18] Shen C. (2019). From machine learning to deep learning: advances in scoring functions for protein–ligand docking. Wiley Interdiscip. Rev. Comput. Mol. Sci..

[bib19] Shen C. (2021). Accuracy or novelty: what can we gain from target-specific machine-learning-based scoring functions in virtual screening?. Briefings Bioinf..

[bib20] Shi D. (2019). Computational insight into the small molecule intervening PD-L1 dimerization and the potential structure-activity relationship. Front. Chem..

[bib21] Tran-Nguyen V.K., Bret G., Rognan D. (2021). True accuracy of fast scoring functions to predict high-throughput screening data from docking poses: the simpler the better. J. Chem. Inf. Model..

[bib22] Upadhaya S., Neftelinov S.T., Hodge J., Campbell J. (2022). Challenges and opportunities in the PD1/PDL1 inhibitor clinical trial landscape. Nat. Rev. Drug Discov..

[bib23] Wójcikowski M., Ballester P.J., Siedlecki P. (2017). Performance of machine-learning scoring functions in structure-based virtual screening. Sci. Rep..

[bib24] Xiong G.-L. (2020). Improving structure-based virtual screening performance via learning from scoring function components. *Brief. Bioinform.* bbaa094.

[bib25] Yasuo N., Sekijima M. (2019). An improved method of structure-based virtual screening via interaction-energy-based learning. J. Chem. Inf. Model..

